# A Successful Therapeutic Challenge: Local Reirradiation of Breast Cancer with a Single Dose of 18 Gy Intraoperative Radiation Therapy (IORT)

**DOI:** 10.7759/cureus.1376

**Published:** 2017-06-20

**Authors:** Elisabetta Bonzano, Marina Guenzi, Renzo Corvò

**Affiliations:** 1 Radiation Oncology, University of Genoa Ospedale Policlinico San Martino; 2 Radiation Oncology, University of Genoa Ospedale Policlinico San Martino

**Keywords:** breast cancer, intraoperative radiotherapy, locoregional recurrence, reirradiation

## Abstract

After standard treatment of primary breast cancer, local relapse can develop in a previously irradiated region. The decision to refer a patient for a second radiotherapy must be thoroughly evaluated due to the increased risk of side effects. We report a case of a 43-year-old Caucasian female with a history of left breast lumpectomy and radiation treatment in 1990 for an invasive ductal carcinoma who presented with a locoregional recurrence 19 years later. After careful evaluation, the patient has undergone a second breast-conserving surgery and successful reirradiation with 18 Gy single dose of intraoperative radiation therapy (IORT).

## Introduction

Nowadays, breast conservative treatment, consisting of conservative surgery followed by adjuvant external whole breast irradiation (WBI), is the main strategy for early breast cancer patients [1-2]. After this approach in early breast cancer patients, the risk of a relapse at 10 years remains about 3% - 5% [3]. When relapse in an irradiated breast occurs, mastectomy is traditionally advised since repeat WBI is not recommended due to the high risk of severe late effects [3]. The aim of this case is to report a potential management adopted to achieve definitive local control in a relapse of breast cancer treated by lumpectomy and intraoperative radiation therapy (IORT).

## Case presentation

On January 25, 1990, a 43-year-old woman underwent left breast conserving surgery with axillary lymph node dissection due to an invasive ductal carcinoma (IDC) pT1bN0 (0/13), 0.7 cm in the largest diameter. After that, she had adjuvant conventional whole breast irradiation with a total dose of 50 Gy in 25 daily fractions delivered over five weeks. Subsequently, she began an annual follow-up by surveillance mammography and ultrasonography. The patient had no evidence of disease until November 2009, when she had a recurrence of IDC, after 19 years of disease-free interval. Tumor size and immunohistochemistry, obtained from the pre-surgical biopsy, were both suitable for a new breast conservative surgical approach with simultaneous IORT. On January 20, 2010, she underwent left lumpectomy and two sentinel lymph nodes were removed for examination. Immediately after tumor resection, an exclusive 18 Gy IORT was performed on the surgical tumor bed of the remaining breast by using a mobile linear accelerator, LIAC (Sordina IORT Technologies S.p.A., Vicenza, Italy), located in the surgical room. A 6 MeV electron energy was chosen, the beam’s collimation was achieved by a 5 cm diameter perspex applicator, and a lead disk (7 cm in diameter) was inserted over the pectoralis major muscle to prevent chest wall irradiation. In vivo dosimetry using a miniature metal oxide semiconductor field effect transistor (micro-MOSFET) was applied. The final pathology demonstrated the tumor size to be 1.5 cm in maximum diameter and the lymph node status was negative. Estrogen receptor (measured by immunohistochemistry) was 96%, progesterone was negative, the Ki67 index was 14%, the human epidermal growth factor receptor-2 (HER-2) profile was negative, and the diagnosis of invasive ductal carcinoma was confirmed. Hormonal therapy was given due to the histological characteristics. The patient was seen for surveillance every six months during the first two years after treatment and once a year thereafter. Now, seven years later, at our most recent follow-up in May of this year, the woman is disease-free and the aesthetic results are excellent with only a mild local fibrosis in the region treated.

## Discussion

Locoregional recurrence after standard treatment of primary breast cancer represents a therapeutic challenge. It has been demonstrated that young age is directly related to the risk of developing a local recurrence, so it is considered an important prognostic factor [4], such as pathologic nodal involvement, and biologic subtype [5]. As stated by the Breast Cancer Expert Panel of the German Society for Radiation Oncology (DEGRO) practice guidelines [6], if an isolated ipsilateral breast tumor recurrence is of limited size (< 2-3 cm), mammography shows unifocal disease, patient age ≥ 50 years, if there is a long interval between primary treatment and recurrence (≥ 48 months), and the patient expresses a preference for a second breast conservation followed by radiotherapy [6], a second breast-conserving approach can be technically feasible. According to the literature, the earlier that the patient has a relapse, the higher the likelihood is that the patient will have a worse overall or disease-free survival [7]. In regards to the management after a local recurrence, there are no standard guidelines [8]. Salvage mastectomy is the most common approach for operable ipsilateral breast relapse. However, a second conservative surgery with partial breast irradiation is being increasingly studied with the aim to improve local control, decreasing toxicity, and avoiding the risks of late effects occurring after a standard second-course whole breast irradiation. In the literature, the most studied approaches are external-beam irradiation and brachytherapy [9]. Our experience, here described, demonstrates that also individualized re-irradiation with IORT is a safe and feasible treatment with long-term local disease control (Table 1).

**Table 1:** Comparison of the Described Case with Cases Treated with External Beam Radiation Therapy (EBRT) Gy: gray; IORT: intraoperative radiation therapy; RT: radiation therapy

	Presented case. Reirradiation with IORT	Deutsch, et al. 2002 [10]. Re- irradiation with EBRT (n = 39)
First RT (Gy) EBRT	50	50 (minimal)
Second RT (Gy)	18 (IORT)	50 (minimal)
Cumulative dose (Gy)	68	100 (minimal)
Interval between RT courses (months)	228	Not available data
Local control	No recurrence at 84 months	68.5% (5-year)
Toxicity	Mild fibrosis	Skin pigmentation changes

## Conclusions

Few data are available in the literature about reirradiation with a single dose of electron beam intraoperative radiotherapy. In a selected group of patients who have developed a locoregional recurrence, treatment with a second breast conservation surgery and IORT appears to be a valid alternative when compared to mastectomy. This approach allows for sparing of the organs at risk and can result in an acceptable cosmetic outcome.
